# Enhancement of adenosine A_2A_ signaling improves dopamine D_2_ receptor antagonist-induced dyskinesia *via* β-arrestin signaling

**DOI:** 10.3389/fnins.2022.1082375

**Published:** 2023-01-24

**Authors:** Koki Nagaoka, Nozomi Asaoka, Kazuki Nagayasu, Hisashi Shirakawa, Shuji Kaneko

**Affiliations:** ^1^Department of Molecular Pharmacology, Graduate School of Pharmaceutical Sciences, Kyoto University, Kyoto, Japan; ^2^Department of Pharmacology, Graduate School of Medicine, Kyoto University, Kyoto, Japan

**Keywords:** adenosine A_2*A*_ receptor, β-arrestin, dopamine D_2_ receptor, indirect pathway medium spiny neuron, tardive dyskinesia

## Abstract

Repeated administration of dopamine D_2_ receptor (D2R) antagonists, which is the treatment for psychosis, often causes tardive dyskinesia (TD). Despite notable clinical demand, effective treatment for TD has not been established yet. The neural mechanism involving the hyperinhibition of indirect pathway medium spiny neurons (iMSNs) in the striatum is considered one of the main causes of TD. In this study, we focused on adenosine A_2A_ receptors (A2ARs) expressed in iMSNs and investigated whether pharmacological activation of A2ARs improves dyskinetic symptoms in a TD mouse model. A 21-day treatment with haloperidol increased the number of vacuous chewing movements (VCMs) and decreased the number of c-Fos^+^/ppENK^+^ iMSNs in the dorsal striatum. Haloperidol-induced VCMs were reduced by acute intraperitoneal administration of an A2AR agonist, CGS 21680A. Consistently, haloperidol-induced VCMs and decrease in the number of c-Fos^+^/ppENK^+^ iMSNs were also mitigated by intrastriatal injection of CGS 21680A. The effects of intrastriatal CGS 21680A were not observed when it was concomitantly administered with a β-arrestin inhibitor, barbadin. Finally, intrastriatal injection of an arrestin-biased D2R agonist, UNC9994, also inhibited haloperidol-induced VCMs. These results suggest that A2AR agonists mitigate TD symptoms by activating striatal iMSNs *via* β-arrestin signaling.

## Introduction

Tardive dyskinesia (TD) is a neurological symptom characterized by involuntary movements of the face, torso, extremities, and sometimes, the respiratory system (Correll et al., 2017). It is considered a late-onset adverse effect induced by long-term use of dopamine D_2_ receptor (D2R) antagonists, such as antipsychotics. TD emerges in about 30% of all patients treated with antipsychotics, and long-term use of D2R antagonists makes TD symptoms irreversible (Carbon et al., 2017). In 2017, the U.S. Food and Drug Administration (FDA) approved valbenazine, an inhibitor of vesicular monoamine transporter 2 (VMAT2), for the treatment of TD; however, the use of VMAT2 inhibitors might cause grave adverse events, such as depression and suicidality (Khorassani et al., 2020). Consequently, the development of a novel anti-dyskinesia drug is imperative but remains challenging.

Striatal dysfunction contributes to motor dysregulation, including dyskinesia (Loonen and Ivanova, 2013). In the striatum, direct pathway medium spiny neurons (dMSNs) express D_1_ receptors (D1Rs) while indirect pathway medium spiny neurons (iMSNs) express D2Rs (Gerfen et al., 1990; Surmeier et al., 1996; Gerfen and Surmeier, 2011), and the balance between iMSN and dMSN activity supports normal motor control (Mink, 2018). Therefore, changes in dMSN and iMSN activity contribute to disease pathology. As hypothesized by Davis and Rosenberg (1979), dopaminergic supersensitivity is widely accepted as a mechanism underlying TD (Margolese et al., 2005). Supporting this hypothesis, animal studies demonstrate that repeated administrations of a D2R antagonist induce upregulation and hypersensitization of D2Rs in the striatum (Burt et al., 1977; Vasconcelos et al., 2003; Samaha et al., 2007) and dyskinetic symptoms (Bordia et al., 2016). Because D2Rs are among the major inhibitory systems in iMSNs, upregulation and hypersensitization of D2Rs might cause hyperinhibition of iMSNs, which may be responsible for TD (Stahl, 2017). Hence, activation of iMSNs may be key to developing novel therapeutic targets for TD.

Adenosine A_2A_ receptors (A2ARs) are expressed in striatal iMSNs (Calabresi et al., 2014). Optogenetic or chemogenetic stimulation of dorsostriatal A2AR-expressing neurons diminishes haloperidol-induced orofacial dyskinesia (Bordia et al., 2016; Nagaoka et al., 2021). Therefore, we focused on A2ARs as a factor involved in the activation of iMSNs.

In the present study, we first examined whether pharmacological activation of A2ARs improves dyskinetic symptoms in a rodent model of haloperidol-induced TD. We then assessed the underlying mechanism using pharmacological approaches.

## Methods

### Animals

All animal experiments were approved by the Kyoto University Animal Research Committee in accordance with the ethical guidelines. Male C57BL/6J mice (6–7 weeks old, 20–30 g) were purchased from Japan SLC (Shizuoka, Japan). All animals were housed at a constant ambient temperature (22 ± 2°C) on a 12-h light/12-h dark cycle, with free access to food and water.

### Drugs and reagents

Haloperidol was purchased from Tokyo Chemical Industry (Tokyo, Japan), CGS 21680A (an A2AR agonist) from Toronto Research Chemicals (Toronto, Canada), and barbadin (a β-arrestin inhibitor; Beautrait et al., 2017) and UNC9994 (an arrestin-biased D2R agonist; Allen et al., 2011) were purchased from Axon MedChem (Groningen, Netherlands).

Haloperidol (1 or 2 mg/kg; Nagaoka et al., 2021) was suspended in 0.5% carboxymethyl cellulose. CGS 21680A, barbadin, and UNC9994 were dissolved in 5% dimethyl sulfoxide with 1% Tween 80 and diluted in saline. The doses for local injection were selected according to their half maximal inhibitory concentration and solubility.

### Haloperidol-induced vacuous chewing movements

Experiments observing vacuous chewing movements (VCMs) were performed as previously described (Nagaoka et al., 2021). Haloperidol was orally administered once daily for 21 days. On the next day after the last dose, the mice were individually placed in a transparent head-fixation chamber (28 mm diameter). The mice were habituated to the chamber for 30 min/day over 3 days and another 60 min immediately before the evaluation. The behavior of the mice was recorded using a video camera placed underneath the mice. The number of VCMs over 3 min was counted thrice in a blinded manner, and the average number of VCMs was analyzed. All behavioral tests were performed during the light phase of the light-dark cycle.

### Microinjection

The guide cannula for microinjection was implanted as previously described (Nagaoka et al., 2021). On the day of the experiment, the injection cannula was inserted into the guide cannula, and the drug (1 ng CGS 21680A, 300 ng barbadin, or 10 ng UNC9994) was injected at a rate of 0.25 μL/min and total injection volume of 1 μL. Subsequently, the injection cannula was left in place during the recording. After the experiments, Evans blue solution (0.5 μL) was injected through the cannula to detect the injection site. If the injection site was incorrect, the animal was excluded from the analysis.

### Repetitive VCM counting

Repetitive VCM experiments were performed as previously described (Nagaoka et al., 2021). In intraperitoneal injection experiments, mice were first administered haloperidol (2 mg/kg/day) orally for 21 days. On day 22, half of the mice were randomly assigned to group 1, which received the vehicle, or group 2, which received intraperitoneally administered CGS 21680A. The mice were individually placed in a transparent head-fixation chamber (28 mm diameter). Beginning from 30 min after drug administration, the behavior of the mice was recorded. The number of VCMs was counted over a 3-min period thrice, and the average number of VCMs was analyzed. Daily haloperidol treatment was continued for 4 more days, and on day 26, VCMs were counted a second time by following a crossover design, i.e., group 1 received CGS 21680A injection while group 2 received vehicle injection.

When counting VCMs with microinjection, mice were orally administered haloperidol (2 mg/kg/day) for 21 days. On day 22, after the basal VCMs were counted once for 5 min, the mice were randomly assigned to groups receiving the vehicle or test drugs through a cannula that was pre-implanted in their dorsal striatum. After 5 min, VCMs were counted again for 5 min to determine the effect of the injected drug. During the crossover study (Figure 2D: vehicle, CGS 21680A, and CGS 21680A + barbadin; Figure 2E: vehicle and barbadin; Figure 2F: vehicle and UNC9994; the order of vehicle or test drug administration to each group was assigned randomly), daily haloperidol treatment was continued for 4 or 8 (when two drugs were co-administered) more days, and on day 26 (or 30), VCMs were counted by following the same procedure followed on day 22. After the experiment, the application sites were identified using Evans blue staining.

**FIGURE 1:**
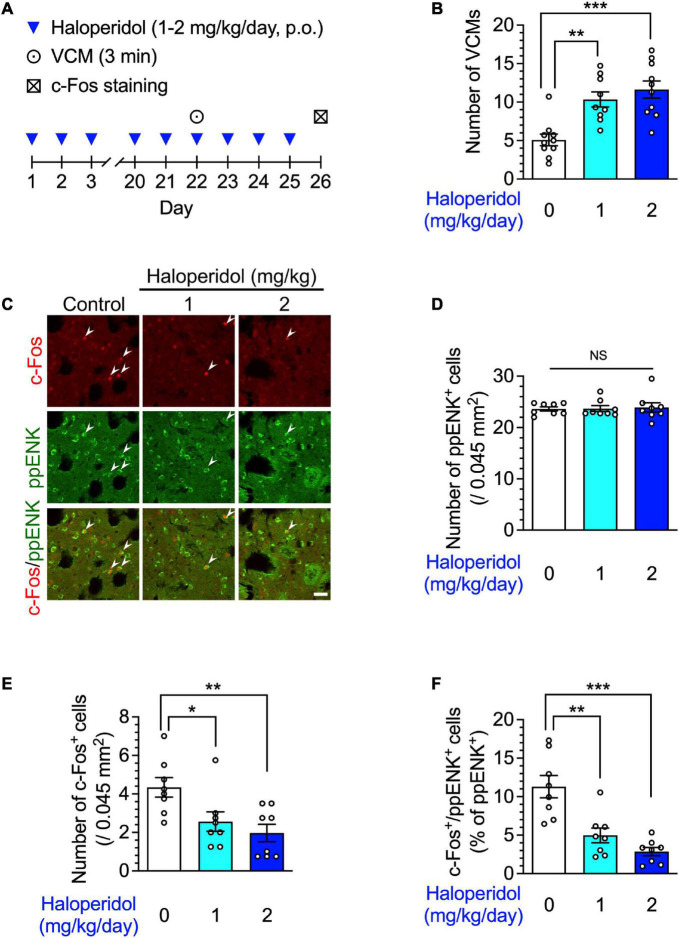
Orofacial dyskinesia and dorsostriatal indirect pathway medium spiny neuron (iMSN) activity in mice after repeated treatment with haloperidol. **(A)** Experimental protocol for counting vacuous chewing movements (VCMs) and immunostaining iMSNs. **(B)** Mice (*n* = 9–10 per group) were treated daily with oral haloperidol (1 or 2 mg/kg/day) for 21 days. The VCMs were counted for 3 min at the 24 h after the last treatment. Statistical significance was tested using one-way analysis of variance (ANOVA) with a *post hoc* Tukey’s test. **P < 0.01; ***P < 0.001. (**C–F**, *n* = 8 per group) After the VCM experiments, the daily haloperidol treatment was continued for 4 more days; on day 26, coronal sections containing the dorsal striatum in mice were prepared, stained with anti-c-Fos and anti-preproenkephalin (ppENK) antibodies, and imaged using confocal microscopy. The number of **(D)** ppENK^+^ cells and **(E)** c-Fos^+^ cells were counted. **(C,F)** The number of c-Fos^+^/ppENK^+^ cells (shown by arrowheads) were counted and are presented as percentages of the number of ppENK^+^ cells, reflecting the total number of iMSNs. Scale bars = 30 μm. Individual data are shown as the mean ± standard error of mean (SEM). Statistical significance was tested using one-way ANOVA with a *post hoc* Tukey’s test. *P < 0.05; **P < 0.01; ***P < 0.001. NS, not significant.

**FIGURE 2:**
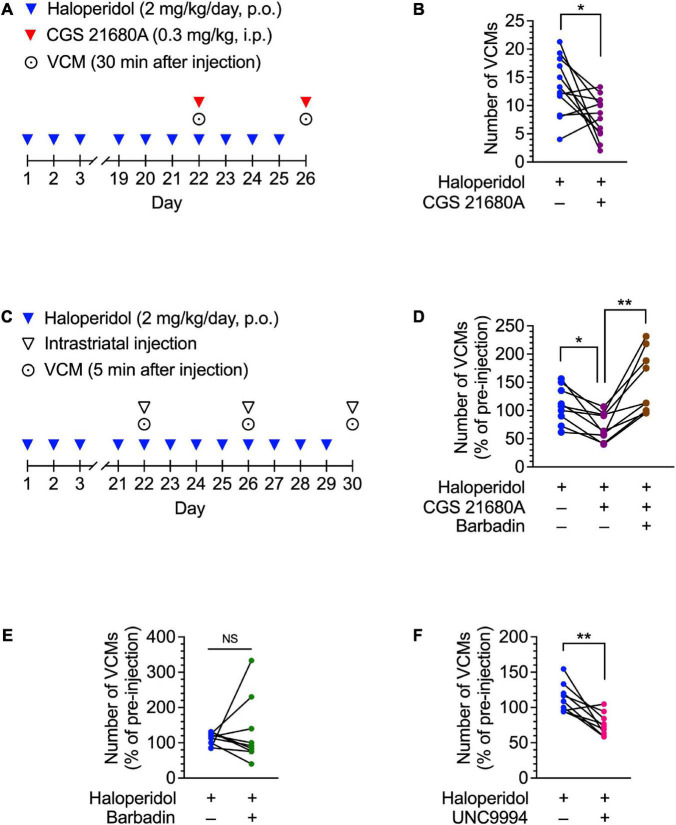
Effects of CGS 21680A, barbadin, and UNC9994 on haloperidol-induced VCMs in mice. **(A,B)** Mice (*n* = 12 per group) were administered oral haloperidol (2 mg/kg/day) daily for 21 days and then CGS 21680A (0.3 mg/kg, i.p.) at 24 h after the last administration of haloperidol. VCMs were counted for 3 min starting at 30 min after CGS 21680A or vehicle administration. Daily haloperidol treatment was continued for 4 more days; on day 26, a second set of VCM measurements were taken in a crossover design. Statistical significance was tested using two-tailed paired *t*-test. *P < 0.05. **(C,D)** Mice (*n* = 9 per group) were orally administered haloperidol (2 mg/kg/day) daily for 21 days. On day 22, VCMs were counted for 5 min as an initial baseline before the mice received vehicle, CGS 21680A (1 ng/side), or CGS 21680A + barbadin (300 ng/side) through a pre-implanted cannula in their dorsal striatum. Five minutes after the injection, the VCMs were counted for another 5 min to determine the effect of the injected drug. Daily haloperidol treatment was continued for 8 more days; on day 26 and 30, the second and third sets of VCM measurements were taken in a crossover design. Changes in the number of VCMs for individual mice on days 22, 26, and 30 are represented as percentages of the pre-injection VCM count. Statistical significance was tested by performing repeated measures one-way ANOVA with a *post hoc* Tukey’s test. *P < 0.05; **P < 0.01. **(E,F)** Mice (*n* = 9 per group) were treated daily with orally administered haloperidol (2 mg/kg/day) for 21 days. On day 22, after the VCMs were counted for 5 min as an initial baseline, half of the mice received vehicle, and the rest received barbadin (300 ng/side) or UNC9994 (10 ng/side) through a pre-implanted cannula in their dorsal striatum. Five minutes later, the number of VCMs was counted for another 5 min to determine the effect of the injected drug. Daily haloperidol treatment was continued for 4 more days; on day 26, the second set of VCM measurements was taken in a crossover design. Changes in the number of VCMs are represented for individual mice on days 22 and 26 as percentages of the pre-injection VCM count. Statistical significance was tested using two-tailed paired *t*-test. **P < 0.01; NS, not significant.

### Immunohistochemistry

Immunohistochemical analysis was performed as previously described (Nagaoka et al., 2021). The mice were anesthetized with pentobarbital and transcardially perfused with phosphate-buffered saline followed by 4% paraformaldehyde (Nacalai Tesque, Kyoto, Japan) in phosphate buffer. In microinjection experiments, perfusion was performed at 65 min after test drug or vehicle injection. The brains were cryosectioned into 30 μm-thick coronal sections using a cryostat (Leica CM3050S; Leica Biosystems, Nussloch, Germany) and stored at –80°C.

To elucidate the c-Fos immunohistochemistry, the sections were immersed in phosphate-buffered saline containing 0.25% Triton-X 100 (Nacalai Tesque) for permeabilization and incubated overnight at room temperature with mouse monoclonal anti-c-Fos antibody (1:500; NBP2-50037, Novus Biologicals) and rabbit monoclonal anti-preproenkephalin (ppENK) antibody (1:500; RA14124, Neuromics, Edina, MN, USA), followed by incubation with Alexa Fluor 594-labeled donkey anti-mouse IgG (1:200; Invitrogen, Waltham, MA, USA) and Alexa Fluor 488-labeled donkey anti-rabbit IgG (1:200; Invitrogen) for 1.5 h at room temperature in the dark. Images were captured using a confocal fluorescence microscope (Fluoview FV10i; Olympus, Tokyo, Japan).

The number of c-Fos^+^/ppENK^+^ cells was counted in four separate fields of the dorsal striatum, 0.3 mm anterior to the bregma; each field covered 0.045 mm^2^. For each batch of confocal images, a uniform background value was set using Adobe Photoshop (Adobe Systems, San Jose, CA, USA). The cells showing ppENK-mediated fluorescence signals above the background level were detected, and c-Fos signals co-localized with DAPI signals were counted.

### Statistics

Statistical analyses using one-way or repeated measures one-way analysis of variance (ANOVA) and two-tailed *t*-test were performed with Prism v9.4.1 (GraphPad Software, La Jolla, CA, USA). For *post hoc* tests, Tukey’s multiple comparisons tests were used. *P* < 0.05 was considered statistically significant. Data are presented as the mean ± standard error of mean (SEM).

## Results

### Repeated administration of haloperidol improves orofacial dyskinetic symptoms and iMSN hypoactivity

We first identified the effects of repeated administration of haloperidol on spontaneous behavior and neuronal activity in the striatum of mice. The mice were administered haloperidol (1 or 2 mg/kg) daily for 21 days, and 24 h after the last treatment, their facial movements were recorded. Consistent with our previous report (Nagaoka et al., 2021), haloperidol-treated mice showed a significant increase in the number of VCMs in a dose-dependent manner (Figures 1A, B, one-way ANOVA: *F*_2,26_ = 13.35, *P* < 0.001; *post hoc* Tukey’s test: ^**^P < 0.01, ^***^P < 0.001).

TD is related to the malfunction of iMSNs within the dorsal striatum (Stahl, 2017). Therefore, we identified iMSNs in the dorsal striatum by detecting immunoreactivity for ppENK, a marker for iMSNs (Gerfen et al., 1990; Grimm et al., 2021) and evaluated the expression of c-Fos protein, which is a product of immediate early genes. After 3 weeks of haloperidol treatment, the number of c-Fos^+^ and c-Fos^+^/ppENK^+^ cells, but not of ppENK^+^ cells, in the dorsal striatum were significantly decreased in a dose-dependent manner (Figures 1A, C–F; one-way ANOVA: Figure 1D, *F*_2,21_ = 0.05, *P* = 0.95; Figure 1E, *F*_2,21_ = 6.41, *P* < 0.01; Figure 1F, *F*_2,21_ = 17.30, *P* < 0.001; *post hoc* Tukey’s test: *P < 0.05, ^**^P < 0.01, ^***^P < 0.001, NS: not significant).

### Intraperitoneal injection of an A2AR agonist inhibited haloperidol-induced dyskinesia

To determine whether pharmacological activation of A2ARs could mitigate D2R antagonist-induced dyskinesia, we intraperitoneally injected a haloperidol-induced VCM mouse model with CGS 21680A, an A2AR agonist. The number of VCMs was repeatedly measured twice on days 22 and 26 in a crossover test (Figure 2A). CGS 21680A (0.3 mg/kg) significantly decreased the number of VCMs, compared with the vehicle-injected control [Figure 2B; paired *t*-test: *t*_(11)_ = 2.73, *P* < 0.05]. These results suggest that acute activation of A2AR is effective in inhibiting orofacial dyskinetic symptoms.

### Intrastriatal injection of an A2AR agonist inhibits haloperidol-induced VCMs *via* β-arrestin signaling

A2ARs activate iMSNs. In iMSNs, A2AR signaling contributes to synaptic plasticity (Shen et al., 2008). Moreover, an A2AR forms a heteromeric complex with a D2R (Zezula and Freissmuth, 2008), and A2AR signaling positively modulates D2R internalization by recruiting β-arrestin to D2Rs (Borroto-Escuela et al., 2011; Huang et al., 2013). Therefore, we hypothesized that stimulation of A2ARs in the dorsal striatum may facilitate D2R internalization *via* β-arrestin, resulting in the inhibition of haloperidol-induced dyskinesia. To test this hypothesis, we examined the effects of intrastriatal injection of CGS 21680A and barbadin, a β-arrestin inhibitor (Beautrait et al., 2017), on haloperidol-induced dyskinesia in a crossover design (Figure 2C). In the haloperidol-induced VCM model mice, the number of VCMs decreased after bilateral infusion of CGS 21680A (1 ng/side), when compared with the vehicle-infused mice, whereas this effect was abolished upon co-administration of CGS 21680A and barbadin (300 ng/side) (Figure 2D; one-way ANOVA: *F*_1.30,10.4_ = 11.73, *P* < 0.01; *post hoc* Tukey’s test: *P < 0.05, ^**^P < 0.01). However, barbadin alone did not affect the number of VCMs in mice after repeated administration of haloperidol [Figure 2E; paired *t*-test: *t*_(8)_ = 0.53, *P* = 0.61]. Additionally, a bilateral intrastriatal infusion of an arrestin-biased D2R agonist UNC9994 (10 ng/side) suppressed haloperidol-induced VCMs [Figure 2F; paired *t*-test: *t*_(8)_ = 3.99, *P* < 0.01], indicating that β-arrestin signaling in the dorsal striatum facilitates the antidyskinetic effect of A2AR activation.

### A2AR activation enhances iMSN activity *via* β-arrestin signaling

**FIGURE 3:**
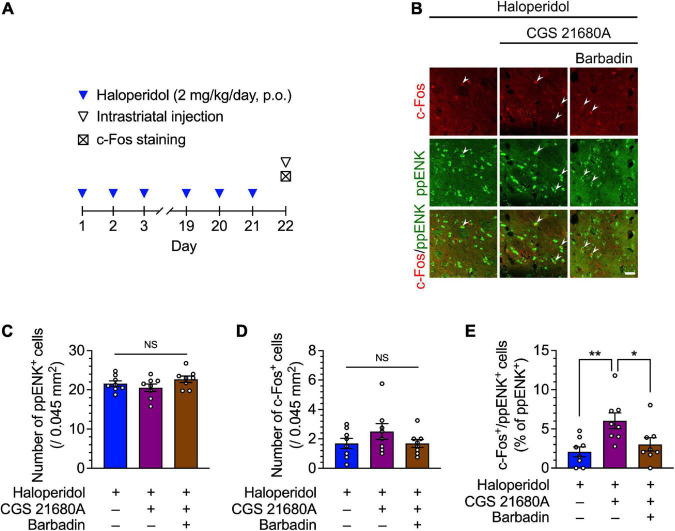
Effects of the dorsostriatal injection of CGS 21680A and barbadin on dorsostriatal iMSN activity in mice after 21 days of treatment with haloperidol. **(A)** Experimental protocol for immunostaining of iMSNs following dorsostriatal injection of CGS 21680A and barbadin. **(B–E)** Mice (*n* = 8 per group) were orally administered haloperidol (2 mg/kg/day) daily for 21 days; 23 h after the last administration of haloperidol, these mice received vehicle, CGS 21680A (1 ng/side), CGS 21680A (1 ng/side) + barbadin (300 ng/side). After 65 min, coronal sections containing the dorsal striatum were prepared, stained with anti-c-Fos and anti-ppENK antibodies, and imaged using confocal microscopy. The number of **(C)** ppENK^+^ cells **(D)** and c-Fos^+^ cells were counted. **(B,E)** The number of c-Fos^+^/ppENK^+^ cells (shown by arrowheads) were counted and are presented as percentages of the number of ppENK^+^ cells, reflecting the total number of iMSNs. Scale bars = 30 μm. Individual data are shown as the mean ± SEM. Statistical significance was tested using one-way ANOVA with a *post hoc* Tukey’s test. *P < 0.05; **P < 0.01. NS, not significant.

Lastly, the neural activity of iMSNs was evaluated after the intrastriatal injection of CGS 21680A, co-administration of CGS 21680A and barbadin, or injection of vehicle in mice that received repeated haloperidol administration (Figure 3A). In the haloperidol-treated mice, no significant difference was observed in the number of ppENK and c-Fos^+^ cells after bilateral infusion with CGS 21680A or co-administration with barbadin (Figures 3B–D; one-way ANOVA: Figure 3C, *F*_2,21_ = 1.71, *P* = 0.21; Figure 3D, *F*_2,21_ = 1.36, *P* = 0.28; *post hoc* Tukey’s test: NS: not significant). The number of c-Fos^+^/ppENK^+^ cells was significantly increased after bilateral infusion with CGS 21680A, whereas this effect of CGS 21680A was abolished by co-administration with barbadin (Figures 3B, E; one-way ANOVA: *F*_2,21_ = 6.09, *P* < 0.01; *post hoc* Tukey’s test: *P < 0.05, ^**^P < 0.01). These results indicate that acute stimulation of A2ARs facilitates the expression of c-Fos protein in the dorsal striatum iMSNs of the VCM model mice, and consistent with the behavior experiments, β-arrestin signaling was required to mitigate the effect of A2AR agonists.

## Discussion

In this study, we characterized D2R antagonist-induced TD-like symptoms and treatment responses in mice. Chronic treatment with haloperidol increased the number of VCMs and decreased the number of c-Fos^+^/ppENK^+^ iMSNs in the dorsal striatum. Intraperitoneal injection of the A2AR agonist, CGS 21680A, acutely decreased haloperidol-induced VCMs. Haloperidol-induced VCMs and decrease in the number of c-Fos^+^/ppENK^+^ iMSNs were inhibited after intrastriatal injection of CGS 21680A, whereas this effect was abolished by co-administration with barbadin. Additionally, the arrestin-biased D2R agonist, UNC9994, inhibited haloperidol-induced VCMs.

A2ARs are expressed at high levels in the iMSN neurons in dorsal and ventral striatum and play an important role in controlling the striatal function (Ciruela et al., 2010). Thus, the A2AR is a potential drug target for striatum-related motor dysfunctions including dyskinesia. In fact, repeated co-administration of A2AR antagonists can prevent L-DOPA-induced dyskinesia (Bibbiani et al., 2003). Bishnoi et al. (2006) reported that repeated co-administration of A2AR antagonist with haloperidol prevents haloperidol-induced orofacial dyskinesia. Thus, these studies suggest the beneficial effect of repeated administration of A2AR antagonists in preventing dyskinetic symptoms induced by chronic alteration of dopaminergic signals. In contrast, the current study revealed the acute therapeutic effect of A2AR agonists on haloperidol-induced dyskinesia. These contradictory findings may be due to the differences in the mechanisms underlying the induction of dyskinesia (repeated administration of haloperidol vs. L-DOPA; Bibbiani et al., 2003) or in the protocols for drug administration (acute administration of A2AR agonist vs. repeated administration of A2AR antagonist; Bishnoi et al., 2006).

The A2AR is a Gs-coupled receptor that activates adenylate cyclase and increases intracellular cAMP. When stimulated, A2ARs enhance the activity of striatal iMSNs by counteracting the Gi/o signaling of D2Rs (Fuxe et al., 2007, 2010). Interestingly, the stimulation of A2ARs in A_2A_-D_2_ heterodimers positively regulates β-arrestin recruitment to D2Rs, resulting in D2R internalization (Borroto-Escuela et al., 2011; Huang et al., 2013), and the functional coupling of heterodimer receptors is impaired in a rat model of Parkinson’s disease (Fernández-Dueñas et al., 2015). Thus, A2AR activation can promote neural activity of iMSNs *via* two pathways, namely, A2AR activation followed by increased intracellular cAMP and β-arrestin-mediated decrease in the number of D2Rs. We examined the effects of intrastriatal injection of CGS 21680A and barbadin, a β-arrestin inhibitor, and observed the decrease in haloperidol-induced VCMs. These findings suggest the contribution of β-arrestin-mediated D2R internalization to the reduction of haloperidol-induced VCMs *via* A2AR stimulation. However, further studies investigating the direct involvement of A2AR-Gs cascade in the induction of iMSN activity are required for understanding the precise therapeutic mechanisms of A2AR stimulation in haloperidol-induced VCMs.

Consistent with the hypothesis that A2AR stimulation recovers the neural activity of iMSNs in haloperidol-treated mice, we showed that repeated administration of haloperidol decreased the number of c-Fos^+^/ppENK^+^ iMSNs, which was successfully increased by intrastriatal activation of A2AR. Because the *c-Fos* gene is known as an immediate early gene that is expressed in response to neural excitation and acts as a transcriptional factor that modulates neural activity (Sheng and Greenberg, 1990), changes in haloperidol- and CGS 21680A-induced *c-Fos* gene expression indicated functional changes in iMSNs. However, based on the results of the immunostaining experiments, we could not completely rule out the possibility that repeated administration of haloperidol affected the signaling pathway between neural excitation and *c-Fos* gene expression, instead of neural excitation itself. Future evaluation of neural excitability will be required to elucidate the underlying mechanisms.

Previous studies showed that acute administration of haloperidol, but not continuous administration, increases striatal D2R availability and thus upregulates c-Fos expression in the striatum (Dragunow et al., 1990; Samaha et al., 2008), suggesting that haloperidol-mediated neural disinhibition is weakened after continuous administration. These observations support the speculation that long-term and/or repeated blockade of D2Rs induce upregulation/hypersensitivity of D2R, which may be a cause of TD (Stahl, 2017). Additionally, both *in vitro* and *in vivo* studies suggest that A2AR stimulation decreases striatal D2R availability (Ferre et al., 1991; Prasad et al., 2022). Taken together, modulation of striatal D2R availability may be involved in the changes in c-Fos expression and VCMs induced by haloperidol and CGS 21680A, although additional studies will be necessary to elucidate the relationship between membrane expression of D2Rs and treatment of haloperidol-induced TD and striatal D2R availability.

The involvement of β-arrestin signaling was supported by the fact that intrastriatal injection of the arrestin-biased D2R agonist UNC9994 acutely mimicked the antidyskinetic effect. UNC9994 acts as a partial agonist on β-arrestin signaling without affecting conventional G protein pathway (Allen et al., 2011). UNC9994 was found to exert an antipsychotic effect only in the presence of A2ARs (Sahlholm et al., 2018), highlighting the role of A_2A_-D_2_ heterodimers in the function of D2Rs. Although further studies are needed, activation of β-arrestin signaling *via* A_2A_-D_2_ heterodimers will be a novel therapeutic target for the treatment of D2R antagonist-induced TD.

In conclusion, we demonstrated that pharmacological activation of A2ARs mitigates dyskinesia induced by long-term administration of D2R antagonists. This study also raises the possibility that β-arrestin recruitment may contribute to the mediation of antidyskinetic effects by stimulating A2ARs.

## Data availability statement

The raw data supporting the conclusions of this article will be made available by the authors, without undue reservation.

## Ethics statement

The animal study was reviewed and approved by the Kyoto University Animal Experiment Committee.

## Author contributions

KoN, NA, KaN, HS, and SK designed the research. KoN performed the research. KoN analyzed the data. KoN, NA, and SK wrote the manuscript. All authors contributed to the article and approved the submitted version.
